# Phenological, Nutritional and Molecular Diversity Assessment among 35 Introduced Lentil (*Lens culinaris* Medik.) Genotypes Grown in Saudi Arabia

**DOI:** 10.3390/ijms15010277

**Published:** 2013-12-27

**Authors:** Salem S. Alghamdi, Altaf M. Khan, Megahed H. Ammar, Ehab H. El-Harty, Hussein M. Migdadi, Samah M. Abd El-Khalik, Aref M. Al-Shameri, Muhammad M. Javed, Sulieman A. Al-Faifi

**Affiliations:** Legume Research Group, Plant production Department, Faculty of Food and Agricultural Sciences, King Saud University, P.O. Box 2460, Riyadh 11451, Saudi Arabia; E-Mails: altaf_sbs@yahoo.com (A.M.K.); ammarrice@gmail.com (M.H.A.); ehabelharty@gmail.com (E.H.E.-H.); smoner@ksu.edu.sa (S.M.A.E.-K.); arefshamiry@yahoo.com (A.M.A.-S.); matloobjaved@gmail.com (M.M.J.); salfaifi@ksu.edu.sa (S.A.A.-F.)

**Keywords:** lentil, nutritional value, molecular markers, genetic diversity

## Abstract

Morphological, nutritional and molecular analyses were carried out to assess genetic diversity among 35 introduced lentil genotypes (*Lens culinaris* Medik.). The genotypes exhibited significant differences for their field parameters and some of them showed noticeable superiority. The nutritional and proximate analysis showed that some genotypes were excellent sources of proteins, essential amino acids, minerals, anti-oxidants, total phenolic contents (TPC) and total flavonoid contents (TFC) and hence, highlights lentil nutritional and medicinal potential. Sequence-related amplified polymorphism (SRAP) and amplified fragments length polymorphism (AFLP) markers were used to estimate the genetic variability at the molecular level. The existence of a considerable amount of genetic diversity among the tested lentil genotypes was also proven at the molecular level. A total of 2894 polymorphic SRAP and 1625 AFLP loci were successfully amplified using six SRAP and four AFLP primer pair combinations. Polymorphism information content (PIC) values for SRAP and AFLP markers were higher than 0.8, indicating the power and higher resolution of those marker systems in detecting molecular diversity. UPGMA (unweighted pair group method with arithmetic average) cluster analysis based on molecular data revealed large number of sub clusters among genotypes, indicating high diversity levels. The data presented here showed that *FLIP2009-64L* and *FLIP2009-69L* could be used as a significant source of yield, total protein, essential amino acids, and antioxidant properties. The results suggest potential lentil cultivation in the central region of Saudi Arabia for its nutritional and medicinal properties, as well as sustainable soil fertility crop.

## 1. Introduction

Lentil (*Lens culinaris* Medik.) is an annual self-pollinated diploid (2× = 2*n* = 14 chromosomes) species belonging to *Legumnosae* (*Fabaceae*) family. It has a relatively large haloid genome size of 4063 Mbp [1]. It is extensively grown in South Asia, Middle East, North Africa, North America and Australia. Lentil world production increased from 3.78 million tonnes (Mt) in 2007 to reach 4.4 million tons in 2011, reflecting its nutritional significance [2]. Lentil seeds contain high protein content, and considered the third-highest level of protein of any legume or nut, after soybeans and hemp. Seed protein content ranges from 22% to 34.6% [3,4]. It also has high levels of carbohydrates (55%–59%) [5,6] and elevated levels of micronutrients and vitamins [7,8]. Besides, the ease of cooking and decortication compared to most other grain legumes may account for increasing global per capita of lentil consumption over the past 50 years, and makes lentil an ideal candidate crop to improve human nutrition through food consumption on a global scale [8,9]. Moreover, owing to its nutritional and medicinal values, low levels of anti-nutrients and an ability to grow in limited water conditions, lentils are potential candidate for conservation agriculture, particularly in Mediterranean arid environments.

Assessing crop genetic variation is vital to understanding the available genetic variability and potential use for varietal improvement [10]. Lentil varieties showed considerable variation for different agro-morphological traits across various environments [11–19]. Morphological variations were used to classify the world collection of lentil germplasm [20].

Advances in molecular biology have led to the development of DNA based markers that can be used for genotype identification, fingerprinting, genetic mapping and diversity assessment [17,21,22]. Several types of DNA markers have been used in lentil genetic diversity assessment and genotyping including: random amplified polymorphic DNA (RAPD) analysis [23–27], inter simple sequence repeat (ISSR) [28–31], simple sequence repeat (SSR) markers [17,32–35], amplified fragment length polymorphism (AFLP) analysis [25,30], restriction fragment length polymorphism (RFLP) [36] and chloroplast DNA [37]. Sequence related amplified polymorphism (SRAP) is a simple and efficient molecular marker technique, with reasonable throughput rate, more reproducible than RAPDs and easier to assay than AFLPs. It can disclose numerous co-dominant markers and, most importantly, SRAP target open reading frames (ORFs) [38]. Amplified fragment length polymorphism (AFLP) markers known as a high potential fingerprinting tool due to its high level of polymorphisms, no prior sequence information is necessary and many markers can be analyzed in a short time. In addition, it is a robust and reliable method because of the stringent reaction conditions for primer annealing [39].

Promoting lentil cultivation in Saudi Arabia will enhance soil fertility and prevent soil desertification by means of areal N_2_ fixation and enrichment of soil organic matter. The present study was undertaken to highlight the potential of lentil cultivation under Saudi Central Region as a promising legume crop. The study also focused on estimating genetic diversity at phenological, chemical and molecular levels.

## 2. Results and Discussion

### 2.1. Phenological Performance

Descriptive values (mean, minimum, maximum, standard deviation and coefficients of variation) of combined data of November 2010 and December 2011 seasons for lentil genotypes are presented in Table 1. Mean performance for lentil genotypes exhibited significant variations for all studied parameters (Tables S1 and S2). Early maturing genotypes were *ILL 3375* with 121.8 days and *FLIP 2011-57L* with 128.8 days. The genotype *FLIP 2009-64L* recorded the highest number of seeds per plant (42.6) and *FLIP 2010-105L* produced the highest seeds yield per plant (1.7 g) compared to the other genotypes. Analysis of variance for vegetative and reproductive traits is presented in Table 2. The results showed high and significant genotypic variance in first, second seasons and their combined data for all studied traits. The first four components in the principal component analysis account for 89.28% of the total variations (Table 3). The first and second principle components demonstrated 33.89% and 24.09% of the total variation respectively. The number of days to 50% flowering, the number of days to 95% maturity, the number of seeds/plant and seed yield/plant explained the maximum variance. The third and fourth component exhibited 16.6% and 14.67% of the total variation where plant height and number of branches/plant traits explained the variation.

### 2.2. Proximate Composition

Descriptive values of lentil genotypes for proximate composition, minerals contents, essentials amino acids, antioxidants and anti-nutritional factors are presented in Table 4. Coefficient of variation varied from 3.49% (moisture content) to 12.11% (ash content). The protein content varied considerably and ranged from 25.3% to 29%. Moisture content in lentil samples ranged from 11.6% to 13.6%. Fat content ranged from 0.79% to 1.19%. Ash content was found to be in the range of 2.39%–2.89% and carbohydrate ranged from 39.5% to 47.0%.

The mineral composition of 35 lentil genotypes showed that potassium and phosphorous content had significant variations among genotypes and ranged from 674.4 to 1061.2 mg/100 g and 286.9 to 546.7 mg/100 g, respectively. Variations in other minerals were also pronounced; magnesium ranged from 126.1 to 157.3 mg/100 g, calcium (64.9 to 84 mg/100 g), iron (6.57 to 8.57 mg/100 g), zinc (2.63 to 4.51 mg/100 g), manganese (1.26 to 2.85 mg/100 g) and copper ranged from 0.86 to 1.37 mg/100 g (Table S3).

Amino acid profiles for the tested lentil genotypes showed also that the essential amino acid Arginine was the most abundant amino acid in most of the lentil genotypes and ranged from 6.6 to 10 g/kg. This was followed by leucine, valine, lysine, phenylalanine, threonine, histidine, and isoleucine which ranged from 6.8 to 9.8, 5.3 to 9.0, 4.5 to 8.6, 5.1 to 8.2, 4.1 to 7.9, 3.6 to 6.1 and 3.4 to 5.6 g/kg, respectively. Tryptophan and methionine were found to be the limiting amino acids in lentil genotypes and ranged from 0.61 to 0.92, and 0.96 to 2.1 g/kg, respectively. Among non-essential amino acids, Glutamic acid was abundant and ranged from 9.1 to 11.9 g/kg. Aspartic acid was the second most abundant amino acids and ranged from 8.2 to 10.9. Other non-essential amino acids, *i.e.*, alanine, glycine, proline, serine and tyrosine, were present in moderate amounts and cysteine was the lowest one and ranged from 0.38 to 0.67 g/kg (Tables S4 and S5).

### 2.3. Anti-Nutritional Factors in Lentil Genotypes

Trypsin inhibitor activity was found to vary from 2.08 in genotype *FLIP 2011-43L* to 2.78 in genotype *FLIP 2011-55L*. Genotypes *ILL 10974*, *ILL 10975*, *FLIP 2011-56L*, *FLIP 2010-100L*, *FLIP 2010-99L*, *FLIP 2009-68L* and *FLIP 2009-70L* showed much higher trypsin inhibitor units (TIU) than any other varieties (Table S6). Tannins content ranged from 0.59 to 0.79 for genotypes *FLIP 2010-106L* and *ILL 10975*, respectively; with an average value 0.68. Phytic acid gave an average of 0.72–1.09 with an average value of 0.92 mg/g. Genotypes *FLIP 2009-70L*, *FLIP 2010-94L*, *FLIP 2009-54L* and *FLIP 2010-97L16* exhibited higher phytic acid values of 1.06, 1.03, 1.01 and 0.96 whereas *FLIP 2009-55L*, *FLIP 2009-51L*, *ILL 3375*, *FLIP 2009-68L* and *FLIP 2010-100L* showed low phytic acid values of 0.72, 0.80, 0.81, 0.81 and 0.82, respectively.

### 2.4. Antioxidant in Lentil Genotypes

The amount of phenolic phytochemicals and antioxidant activity of the 35 selected lentil genotypes showed that the total phenolic components (TPC) ranged from 5.7 to 20.8 mg GAE (galic acid equivalent)/g. Lentil genotypes *FLIP 2010-96L* (20.82 mg GAE/g), *ILL 10974* (20.52 mg GAE/g), and *FLIP 2010-95L* (20.01 mg GAE/g) had higher phenolic content. On the other hand, *ILL 10975* recorded the lowest value of 6.45 mg GAE/g. Flavonoid content for lentil seeds are tabulated (Table S7). Lentil genotype *ILL3375* recorded lowest flavonoid content [4.12 mg QE (Quercetin Equivalent)/g dry seed] and *ILL10974* recorded highest value (8.92 mg QE/g seed). Antioxidant activity measured as the effect of phenolic compounds on the Table S8, 2-diphenyl-1-picryl hydrazyl radical (DPPH) activity ranged from 10.61 to 23.26 μg/g for *FLIP 2011-54L* and *ILL 10974*, respectively (Table S6).

### 2.5. Genetic Diversity Based on SRAP and AFLP Markers

**Figure 1.**
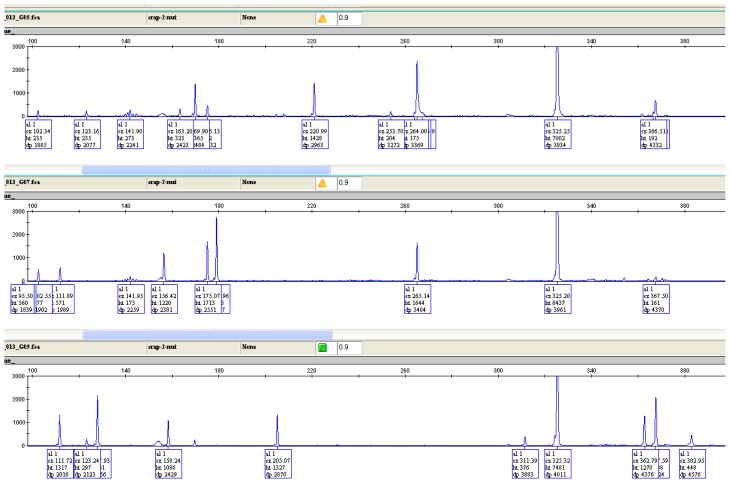
SRAP markers electropherograms of six lentil genotypes using ME2/EM2 primer combination analyzed in the GeneMapper software (Applied Biosystems, Foster City, CA, USA).

Using six SRAP primer combinations, the 35 genotypes of lentil produced 2894 amplified products with an average of 482 bands/primer across all genotypes (Table 7). All the scored bands were polymorphic. Figure 1 showed the peak patterns of the tested genotypes using SRAP primer combination ME2/EM2. Primer combination ME2/EM6 produced the highest number of fragments (1138 fragments) and primer combination ME2/EM4 produced the lowest number of fragments with only 124 fragments. PIC values measured for primer combinations were high and ranged from 0.908 to 0.984. The high PIC values reflected the power of SRAP markers to discriminate lentil genotypes and assess genetic diversity.

**Figure 2.**
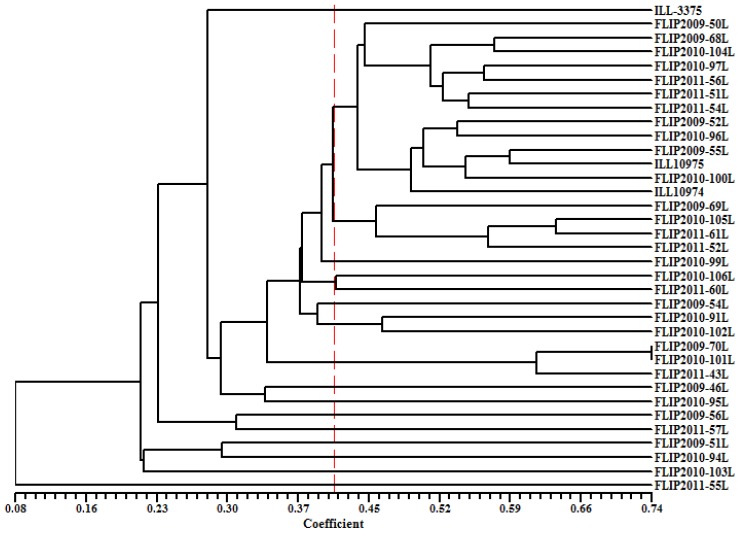
Dendrogram of 35 lentil genotypes generated from Sequence-related amplified polymorphism SRAP markers by Jaccard’s coefficient and UPGMA clustering method. Similarity values are shown at bottom of dendrogram.

Genetic similarity matrix among all studied genotype pairs using Jaccard coefficients is presented in Figures S1 and S2. The highest similarity was found between genotype pair *FLIP 2009-70L* and *FLIP 2010-101L* (74%). A dendrogram constructed to explain genetic relationship using SRAP markers based on UPGMA method is presented in Figure 2. The data revealed significant genetic differences as similarities ranged from 8% to 74%. The genotype *FLIP 2011-55L* failed to form any cluster and was considered the most divergent genotype. The remaining genotypes were grouped in two main clusters (A and B) at 21% similarity. Group A contained only three genotypes *FLIP 2010-94L*, *FLIP 2010-103L* and *FLIP 2010-94L* and further separated into individual genotypes at 30% similarity level. The group B contained 31 genotypes and further divided into many sub-groups at 30% similarity, confirming the existence of a considerable amount of genetic diversity among the lentil genotypes at the molecular level.

A total of 1625 amplified bands were obtained by four AFLP primer combinations with an average of 406 bands/primer combination (Table 5). Primer combination E_TC_/M_CTA_ produced the highest number of fragments which were 583 while primer combination E_CC_/M_CCT_ produced the lowest number of fragments (98 fragments only). PIC values measured for individual primer combination ranged from 0.894 to 0.968 showing high resolving power of AFLP markers.

**Figure 3.**
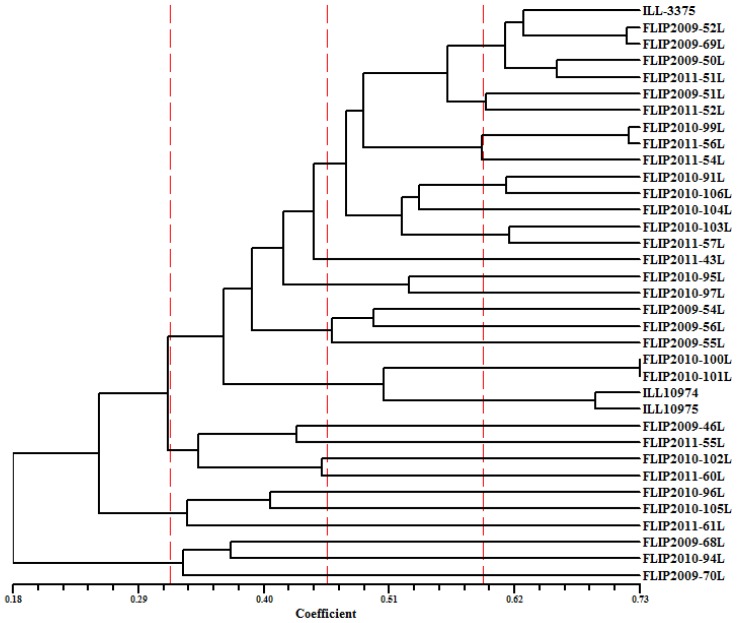
Dendrogram of 35 lentil genotypes generated from Amplified fragments length polymorphism AFLP markers by Jacc, ard’s coefficient and UPGMA (unweighted pair group method with arithmetic average) clustering method. Similarity values are shown at bottom of dendrogram.

Genetic similarity matrix among studied genotypes by four AFLP primer combinations using Jaccard coefficients showed highest similarity between genotype pairs *FLIP 2010-99L* and *FLIP 2011-56L* (68%). The dendrogram constructed based on UPGMA (unweighted pair group method with arithmetic average) clustering method was used for detecting genetic diversity of genotypes (Figure 3). Cluster analysis using AFLP data was followed the SRAP data patterns. The clustering divided the 35 genotypes into two main groups (A and B). Group A contains genotypes named as *FLIP 2009-70L*, *FLIP 2010-94L* and *FLIP 2009-68L*. Group B contains all other genotypes and further divided into several sub-clusters at 43% similarity level. The high number of subgroups reflected the variability in studied genotypes, and marker resolution power as well.

### 2.6. Discussion

Germplasm genetic variability assessment with phenological parameters provides the basis for adaptation to the climatic variables of the prevailing environment. In this study, 35 lentil genotypes showed highly significant morphological variations suggesting that the genotypes have potential and warranty in breeding programs. These results were consistent with those reported by [14,18,19,40–49].

Principal component analysis showed that the first four principal components (PCs) were more significant as they accounted for 89.28% of total variations. Days to flowering and seed yield in PC1 and number of seed/plant in PC2 were the most important traits and these were the most interrelated variables with each other, indicating great scope for improvement in these traits through selection. These results agree with those of [44,50] who reported that days to flowering and maturity was positively correlated with seed yield in lentil.

This PC analysis suggests that seed yield and days to flowering could be the main selection criteria, since they displayed a large amount of the variability in each PC. From the analyses of variance, all traits revealed significant variation among genotypes except for number of branches. These results are in agreement with that of [49] who found no significant difference for branch production among the mutants/mother variety. Significant variation in number of seeds per pod [51] and 100-seed weight [52] was also reported. It was reported also that [53] seed yield in lentil depends on seed size, and seed yield increased with increased number of pods per plant [50,54–57]. The crude protein results are almost consistent with those reported by [58], who obtained 26% in lentil genotypes; however, [59] estimated crude protein for green and red lentils was 23.03% and 25.88%, respectively. Moreover, [60] reported around 25% protein content in lentils, and [61] reported between 24.3% and 30.2% protein contents. Moisture content ranged between 11.6% and 13.6%. Carbohydrate content ranged from 39.5% to 47% and was close to that reported earlier [62–64]. Ash content was found to be in the range of 2.39%–2.89% which was in line with values described by [65,66]. Fat content ranged from 0.79% to 1.19% which was lower than those described by [62]. However, our results were more or less close to that of [66,67] who reported 1%–2% lipid content in lentil seeds.

All lentil genotypes contained relatively high amounts of calcium, potassium, phosphorous, zinc, and magnesium, manganese, iron and copper and thus, could be potential sources for human mineral requirements. The mineral contents of lentil varieties in our study were in harmony with those reported by other researchers [68–71]. The amino acid composition of the lentil genotypes indicated significant variation in essential amino acids. The results obtained were fairly comparable with those reported by [58,59]. Generally, the chemical score and amino acid index are used for screening potential protein foods with reference to the FAO/WHO standard amino acid profiles established for humans [72]. The results indicate that all essential amino acids, except methionine and tryptophan, were present in considerable amounts in all the genotypes analyzed.

Antioxidants are important in reducing oxidative damage associated with many diseases, including cardiovascular disease, cancer, atherosclerosis, diabetes, immune deficiency diseases and ageing [73]. Total antioxidant activities are significantly correlated with total phenolic content (TPC) [74], and the bioavailability of polyphenols [75]. The proton radical scavenging action is known to be one of the various mechanisms for measuring antioxidant activity. DPPH is one of the compounds that possess a proton free radical and shows a maximum absorption at 517 nm. This assay determines the scavenging of stable radical species of DPPH by antioxidants. The results obtained here showed potential of lentil scavenging capacity and hence the power of free radicals elimination when incorporated in diet. Similar results were reported by [75–77]. They highlighted the significance of the free radical scavenging capacity of lentil genotypes and proved their medical potential.

Good knowledge of the different DNA-based markers is an important step for plant germplasm characterization and classification, and a prerequisite for their effective application in breeding programs [78]. SRAP and AFLP markers proved to be highly efficient tools in discriminating between the 35 lentils genotypes analyzed. The number of polymorphic bands/primer reported here was higher than that reported earlier. For instance, [30] detected lower AFLP polymorphic fragments using Turkish lentil landraces and cultivars (19.8 fragments/primer). Average PIC values of 0.952 for SRAP and 0.945 for AFLP were obtained. These values were much higher than those reported for other marker systems [30]. The higher number of polymorphic fragments in the current study could be due to the higher resolution power and sensitivity of the fragment detection system used.

The comparison of the two molecular marker systems used indicated similar PIC values in AFLP and SRAP markers. Therefore, both markers systems could be recommended for assessing molecular diversity, fingerprinting and varietal discrimination. Both markers revealed a high degree of similarity in dendrogram topology, though there are little differences in some genotype positions in sub-clusters. Overall, genotypes were tending to cluster according to their origin and sometimes a few genotypes correlated with their genetic background.

Our results demonstrated that SRAP and AFLP markers were very useful for genetic classification in lentil genotypes. Nei dissimilarity matrices of AFLP and SRAP markers were compared using Mantel test. The results revealed that SRAP and AFLP markers were correlated (*r* = 0.189). Similar results were reported on AFLP and ISSR for genetic variation among Turkey lentil landraces [30], where significant a correlation coefficient was obtained using the Mantel test.

## 3. Experimental Section

Thirty five advanced breeding lines of lentils were introduced from International Center for Agricultural Research in Dry Areas (ICARDA) and used for this study. The pedigree and geographical origins of the studied genotypes are presented in Table 6. Seeds were grown at Dirab Experimental Research Station, Riyadh, Saudi Arabia (24°43′34″N, 46°37′15″E) in randomized complete block design (RCBD) layout with three replications in November 2010 and December 2011 growing seasons for field evaluation. Seeds were sown in mid-November using a hand drill. The experimental plot consisted of two rows, 30 cm apart and 3 m long (1.8 m^2^ size). Data were recorded on phenological parameters, including seed yield and its components. The dry seeds proximate analyses of the genotypes for crude proteins, moisture, total ash and crude fat were carried out in triplicate using the methods described in [79]. All the proximate values were reported in g/100 g seed dry weight. Carbohydrate was determined using subtraction. Percentage of nitrogen in seed samples were measured and crude protein% was calculated as nitrogen% × 6.25 [80]. Minerals content were determined according to [81]. About 100–500 μg protein sample was completely hydrolysed in HCl at 110 °C for 24 h, gradient HPLC system LC-10AT vp (Shimadzu corporation, Kyoto, Japan) with auto injector was used to determine amino acids profiles. Estimation of antioxidant activities of seed samples were also carried out. Total phenolic compounds were quantified by Folin-Ciocalteu method, according to [82]. Colorimetric aluminum chloride method was used for flavonoid determination as described by [82]. The effect of phenolic compounds on the Table S8, 2-diphenyl-1-picryl hydrazyl radical (DPPH) were used to assess antioxidant activity of the extracts. The ability of the samples to scavenge DPPH radicals was determined according to [83]. The values of total phenolic components (TPC), total flavonoid content (TFC) and DPPH (mg standard equivalent per gram of dry weight) were estimated using the equation:

(1) $$\text{TPC},\text{TFC or DPPH}\;\text{in }(\text{mg standard equivalent per gram}\;\text{weight})=\frac{\left[(A\text{s}-A\text{b})(\text{Slope})](20/V\right)}{I\times 1000}$$

where: *A*s = sample absorbance for TPC, TFC or absorbance decrease of sample for DPPH value; *A*b = blank (no extract) absorbance for TPC and TFC or absorbance decrease of blank for DPPH value (extract was substituted by deionized water for blank); Slope = slope of standard curve, (20/*V*) = total volume of extract (20 mL)/used volume of extract (mL); *I* = weight of sample used (g); 1000 = factor for changing μg to mg.

Trypsin inhibitor activity was assessed according to [84] using benzoyl-dl-arginine-*p*-nitroanilide (BAPA) as synthetic substrate. Trypsin inhibitor unit per milligram (TIU/mg) of sample was calculated by taking absorbance at 410 nm against a blank in a spectrometer. One unit of TIU is defined as an increase of 0.01 in absorbance reading at 410 nm per 10 mL of reaction mixture. Phytic acid analysis was performed according to [85] using chromophore reagent. Tannin content was determined using the vanillin-HCl method of [86].

For molecular characterization, two-week old lentil leaves from 35 selected genotypes were collected, dropped in liquid N_2_, and stored at −80 °C until DNA isolation. DNA isolation was carried out using a modified SDS protocol [87] as described by [88]. Six SRAP and four AFLP primer combinations were used to estimate genetic diversity among lentil genotypes. The SRAP primer combinations used are shown in Table 7. SRAP-PCR reactions were performed in 20 μL volume containing 1× *GoTaq* Green Master Mix (Cat. No. M7123, Promega Corporation, Madison, WI, USA), 0.25 μM from each forward and reveries primers, 50 ng template DNA and nuclease-free water up to 20 μL. The forward primers were 5′ end labelled with FAM dye (Applied Biosystems, Foster city, CA, USA). Amplification was carried out on a TC-5000 thermal cycler (Bibby Scientific, Staffordshire, UK) as follows: initial denaturation at 94 °C for 5 min followed by five cycles of denaturing at 94 °C for 1 min, annealing at 35 °C for 1 min and elongation at 72 °C for 1 min. In the remaining 30 cycles, the annealing temperature was increased to 50 °C for 1 min with a final extension step at 72 °C for 7 min. AFLP analysis was performed following the procedure in PE Biosystems plant mapping kit (Applied Biosystems, Foster City, CA, USA) using a modified procedure from [39] and four *Eco*RI/*Mse*I primer combinations, *i.e.*, E_CT_/M_CTG_, E_TA_/M_CTC_, E_TC_/M_CTA_, and E_CC_/M_CCT_. One microliter of the PCR amplified product was mixed with 0.05 μL of the GeneScan 500 LIZ size standard (Applied Biosystems P/N 4322682) and 9 μL of Hi-Di Formamide (Applied Biosystems P/N 4311320). The mixture was denatured for 3 min at 95 °C and loaded on the 36-cm 16-capillary system of the Applied Biosystems 3130*xl* Genetic Analyzer. Fragment analysis for SRAP and AFLP was performed with GeneMapper Analysis Software v3.7 (Applied Biosystems) and the data were assembled in binary format allele presence (1) or (0) for Absence. The threshold for allele calling was set at 200 relative florescence units (rfu) according to [89]. Fragment analysis was carried out for allele sizes in the range of 100–500 bp. Markers showed single alleles across genotypes were eliminated from the analysis. Data generating from SRAP and AFLP analysis were analysed using Jaccard similarity coefficient [90]. Dendrogram was constructed using Jaccard similarity coefficient and the unweighted pair group method with arithmetic average (UPGMA) employing the SAHN (sequential, agglomerative, hierarchical, and nested clustering) from the NTSYSpc (ver.2.10) program (Exeter Publishing Ltd., New York, NY, USA) [91]. Analysis of variance (ANOVA) was carried out using *MSTATC* software (Michigan State University, East Lansing, MI, USA) and principal component analysis using *XLSTAT* software (Addin Soft, New York, NY, USA).

## 4. Conclusions

In conclusion, analysis on phenological, nutritional and molecular data was useful for assessing genetic diversity in lentils. The lentil genotypes demonstrated significant differences and some genotypes showed superiority in most of the studied parameters. Overall, the five best performing genotypes among the lentils were *FLIP 2009-64L*, *FLIP 2009-69L*, *FLIP 2010-94L*, *FLIP 2010-101L*, *FLIP 2010-102 L*, *FLIP 2010-104 L* and *FLIP 2010-105 L*. These genotypes, along with other high yielding genotypes, are suggested for lentil genetic improvement. Incorporation of lentils in daily human diets can thus enhance nutritional status and reduce malnutrition. Their high protein content with elevated levels of essential amino acids, along with high nutrients and antioxidants levels, makes lentil seeds an ideal meat alternative. SRAP and AFLP marker systems showed high levels of resolution and discrimination power, making them ideal systems for assessing genetic diversity and varietal discrimination.

## Supplementary Information



## Figures and Tables

**Table 1.** Mean, minimum and maximum values, standard deviation (SD) and coefficient of variability (CV) of vegetative and reproductive studied traits for combined data of the two seasons (2011 and 2012) for 35 lentil studied genotypes.

Character	Mean	SD	Min	Max	CV
Number of days to 50% flowering	102.8	5.26	86	112	5.1
Number of days to 95% maturing	135.8	4.66	121.8	142.6	3.4
Plant height (cm)	31	2.34	27	35.7	7.5
Number of branches/plant	3.25	0.497	2.4	4.3	15.2
Number of seeds/plant	28.4	5.64	18.8	42.6	19.8
Seed yield/plant	1.09	0.222	0.8	1.5	20.3

**Table 2.** Combined mean squares estimates for studied traits. S.O.V (source of variation); DF (Degrees of Freedom); No. (Number).

S.O.V	DF	No. of Days to 50% Flowering	No. of Days to 95% Maturing	Plant Height (cm)	No. of Branches	No. of Seeds/Plant	Seed Yield/Plant (g)
Year (Y)	1	2597.1 **	5260.0 **	6956.9 **	3.55 ns	6296.5 **	7.13 **
Error	4	15.7	10.3	14.1	1.2	1.2	0.04
Genotype (G)	34	166.2 **	130.5 **	33.0 **	1.50 **	191.6 **	0.31 **
G × Y	34	14.9 ns	20.8 **	32.3 **	0.47 ns	46.9 ns	0.06 ns
Error	136	17.5	11.4	14.9	0.6	34.6	0.07

** Highly significant (*p* < 0.001);

ns, not significant.

**Table 3.** Eigen values, individual and cumulative percentage variations and eigen vectors explained by four principal components based on morphological traits in 35 lentil genotypes.

Components	P1	P2	P3	P4
Eigen value	2.034	1.446	0.997	0.881
Variability (%)	33.894	24.092	16.616	14.679
Cumulative (%)	33.894	57.986	74.602	89.281
*Eigen Vectors*				
Days to 50% Flowering	0.502	−0.467	−0.049	0.24
Days to 95% Maturity	0.571	−0.313	0.139	0.235
Plant Height	−0.028	−0.333	0.718	−0.604
Number of Branches/Plant	−0.064	0.361	0.674	0.625
Number of Seeds/Plant	0.432	0.503	0.073	−0.271
Seed Yield/Plant	0.481	0.436	−0.048	−0.239

**Table 4.** Mean, minimum values, maximum values, standard deviation (SD) and coefficient of variability (CV) of proximate analysis in lentil genotypes (on dry weight basis).

Variable	Mean	SD	CV	Min	Max
Crude protein (%)	27.346	1.273	4.66	25.3	29.3
Moisture (%)	12.631	0.44	3.49	11.6	13.6
Ash (%)	2.60	0.115	4.42	2.39	2.89
Fats (%)	0.9991	0.121	12.11	0.72	1.19
Carbohydrate (%)	43.997	2.215	5.03	39.5	47.1
*Essentials Amino Acids (g/kg)*					
Leu	8.643	0.797	9.22	6.8	9.8
Ile	4.569	0.636	13.92	3.4	5.6
Phe	6.389	0.875	13.7	4.9	8.2
Try	0.7909	0.091	11.5	0.61	0.92
His	4.84	0.63	13.02	3.6	6.1
Val	7.389	0.991	13.42	5.3	9
Thr	6.011	1.087	18.08	4.1	7.9
Met	1.5031	0.3127	20.8	0.85	2.1
Lys	7.309	1.018	13.94	4.5	8.6
Arg	8.526	1.001	11.74	6.6	10
*Minerals (mg/100 g)*					
Ca	74.92	4.433	5.92	64.9	84.8
P	412.4	66.5	16.13	286.9	546.7
K	886.1	99.6	11.24	674.4	1061.2
Mg	138.58	6.98	5.04	126.1	157.3
Fe	7.4483	0.4455	5.98	6.57	8.57
Mn	1.5317	0.3818	24.92	1.26	2.85
Cu	1.1337	0.1617	14.26	0.86	1.37
Zn	3.7777	0.4809	12.73	2.63	4.51
*Antioxidants*					
TPC a (mg GAE/g)	13.467	4.546	33.76	6.45	20.82
TFC b (mg QE/g)	6.03	1.38	22.89	4.12	8.92
DPPH c (μg/g)	15.092	3.036	20.12	10.61	23.26
*Anti-nutritional Factors*					
Protease inhibitors (mg/g)	2.5234	0.1847	7.32	2.08	2.78
Tannins (mg/g)	0.6846	0.068	9.93	0.52	0.79
Phytic acids (mg/g)	0.928	0.0908	9.79	0.72	1.09

a Total phenolic components calculated as galic acid equivalent/g seed;

b total flavenoid content (mg Quercetin Equivalent/g seed);

c 2-diphenyl-1-picryl hydrazyl radical (μg/g).

**Table 5.** Summary of sequence-related amplified polymorphism (SRAP) and amplified fragments length polymorphism (AFLP) primers combinations results. PIC: polymorphic information content.

SRAP					AFLP				
	
Primer Combination	Total Fragments	Polymorphic Fragments	Polymorphism Rate (%)	PIC	Primer Combination	Total Fragments	Polymorphic Fragments	Polymorphism Rate (%)	PIC
ME1/EM1	132	132	100	0.924	E_CT_/M_CTG_	569	569	100	0.968
ME1/EM2	393	393	100	0.964	E_TA_/M_CTC_	375	375	100	0.957
ME2/EM3	399	399	100	0.963	E_TC_/M_CTA_	583	583	100	0.963
ME2/EM4	124	124	100	0.908	E_CC_/M_CCT_	98	98	100	0.894
ME2/EM5	708	708	100	0.970	Total	1625	-	-	-
ME2/EM6	1138	1138	100	0.984	Mean	406.25	-	-	-
Total (Mean)	2894 (482)	-	-	-	-	-	-	-	-

**Table 6.** Pedigree and origin of 35 introduced lentil genotypes used. ICARDA: International Center for Agricultural Research in Dry Areas.

Genotype	Pedigree	Origin
*ILL 3375*	*ILL 3375*	India
*FLIP2009-50L*	*ILL 8090* × *ILL 7685*	ICARDA
*FLIP 2009-51L*	*ILL 7617* × *ILL 4404*	ICARDA
*FLIP 2009-52L*	*ILL 5883* × *ILL 8113*	ICARDA
*FLIP 2009-54L*	*ILL 7012* × *ILL 4404*	ICARDA
*FLIP 2009-55L*	*ILL 6783* × *ILL 98*	ICARDA
*FLIP 2009-56L*	*ILL 8077* × *ILL 6994*	ICARDA
*FLIP 2009-64L*	-	ICARDA
*FLIP 2009-68L*	*ILL 7713* × *ILL 7201*	ICARDA
*FLIP 2009-69L*	*ILL 790* × *ILL 7706*	ICARDA
*FLIP 2009-70L*	*ILL 7537* × *ILL 4404*	ICARDA
*FLIP 2010-91L*	*ILL 8114* × *ILL 7555*	ICARDA
*FLIP 2010-94L*	*ILL 7620* × *ILL 8113*	ICARDA
*FLIP 2010-95L*	*ILL 7620* × *ILL 8113*	ICARDA
*FLIP 2010-96L*	*ILL 7620* × *ILL 8113*	ICARDA
*FLIP 2010-97L*	*ILL 7620* × *ILL 8113*	ICARDA
*FLIP 2010-99L*	*ILL 7620* × *ILL 8113*	ICARDA
*FLIP2010-100L*	*ILL 2501* × *ILL 7537*	ICARDA
*FLIP 2010-101L*	*ILL 2501* × *ILL 7537*	ICARDA
*FLIP 2010-102L*	*ILL 4402* × *ILL 2501*	ICARDA
*FLIP 2010-103L*	*ILL 358* × *ILL 87062*	ICARDA
*FLIP 2010-104L*	*ILL 6037* × *ILL 87062*	ICARDA
*FLIP 2010-105L*	*ILL 7723* × *ILL 87062*	ICARDA
*FLIP 2010-106L*	*ILL 7723* × *ILL 87062*	ICARDA
*FLIP 2011-43L*	*ILL 7537* × *ILL 590*	ICARDA
*FLIP 2011-51L*	*ILL 590* × *ILL 7979*	ICARDA
*FLIP 2011-52L*	*ILL 7010* × *ILL 6971*	ICARDA
*FLIP 2011-54L*	*ILL 8090* × *ILL 7980*	ICARDA
*FLIP 2011-55L*	*ILL 8090* × *ILL 7980*	ICARDA
*FLIP 2011-56L*	*ILL 8090* × *ILL 7980*	ICARDA
*FLIP 2011-57L*	*ILL 8090* × *ILL 7980*	ICARDA
*ILL 10974*	*96-024L* × *99H046*	Australia
*ILL 10975*	*97-011* × *98H006-99HS001*	Australia
*FLIP 2011-60L*	*ILL7723* × *ILL8090*	ICARDA
*FLIP 2011-61L*	*ILL7537* × *ILL590*	ICARDA
-	-	-

**Table 7.** Name and sequence of SRAP primers used.

Primer name	Forward 5′–3′	Primer name	Reverse 5′–3′
ME1	TGAGTCCAAACCGGATA	EM1	GACTGCGTACGAATTAAT
ME2	TGAGTCCAAACCGGAGC	EM2	GACTGCGTACGAATTTGC
ME3	TGAGTCCAAACCGGAAT	EM3	GACTGCGTACGAATTGAC
