# Supplementary material for: Phenological, Nutritional and Molecular Diversity Assessment among 35 Introduced Lentil (Lens culinaris Medik.) Genotypes Grown in Saudi Arabia
Source: Int J Mol Sci. 2013 Dec 27;15(1):277–95. doi: 10.3390/ijms15010277 (PMC3907810; doi:10.3390/ijms15010277)
Supplement: Supplementary file 1 [file ijms-15-00277-s001.pdf]

Supplementary Information

Table S1. Vegetative Performance of 35 lentil genotypes grown at Dirab Agriculture Station (Riyadh, Saudi Arabia) in November 2010, December 2011 and combined of the two seasons.

Genotype	Number of Days to 50% Flowering			Number of Days to 95% Maturing			Plant Height (cm)		
	2010	2011	Combined	2010	2011	Combined	2010	2011	Combined
ILL 3375	89.7	82.3	86.0	130.0	113.7	121.8	38.2	22.7	30.5
FLIP2009-50L	97.0	90.0	93.5	134.8	123.8	129.3	34.8	20.9	27.8
FLIP 2009-51L	111.4	105.4	108.4	143.3	130.5	136.9	34.5	25.8	30.2
FLIP 2009-52L	111.3	101.0	106.2	144.9	133.6	139.3	38.5	24.0	31.3
FLIP 2009-54L	108.7	107.7	108.2	142.2	136.4	139.3	34.5	32.9	33.7
FLIP 2009-55L	106.2	100.1	103.1	139.2	133.5	136.4	31.8	35.0	33.4
FLIP 2009-56L	100.7	102.7	101.7	134.4	141.6	138.0	32.0	33.3	32.6
FLIP 2009-64L	109.3	101.0	105.2	144.0	138.0	141.0	40.0	27.0	33.5
FLIP 2009-68L	102.2	95.2	98.7	143.6	132.6	138.1	35.3	23.3	29.3
FLIP 2009-69L	105.0	96.3	100.7	137.1	127.8	132.4	33.8	22.2	28.0
FLIP 2009-70L	103.7	98.7	101.2	136.3	125.3	130.8	37.3	24.2	30.8
FLIP 2010-91L	110.0	103.0	106.5	138.6	128.9	133.8	32.1	22.4	27.2
FLIP 2010-94L	101.0	93.0	97.0	133.5	124.5	129.0	36.1	24.1	30.1
FLIP 2010-95L	113.1	100.0	106.6	142.5	131.5	137.0	35.1	23.1	29.1
FLIP 2010-96L	106.0	98.0	102.0	147.3	136.3	141.8	39.5	27.5	33.5
FLIP 2010-97L	107.3	98.7	103.0	136.2	125.2	130.7	36.1	24.1	30.1
FLIP 2010-99L	98.4	91.4	94.9	146.3	135.3	140.8	36.1	25.8	30.9
FLIP 2010-100L	112.3	102.7	107.5	148.1	137.1	142.6	36.9	27.3	32.1
FLIP 2010-101L	112.0	105.0	108.5	148.1	137.1	142.6	34.0	22.0	28.0
FLIP 2010-102L	109.9	101.0	105.5	145.2	133.2	139.2	37.1	24.1	30.6
FLIP 2010-103L	107.0	95.7	101.3	142.1	132.8	137.4	34.0	20.0	27.0
FLIP 2010-104L	101.0	96.3	98.7	137.1	128.8	132.9	37.1	24.8	30.9

Table S1. *Cont.*

Genotype	Number of Days to 50% Flowering			Number of Days to 95% Maturing			Plant Height (cm)		
	**2010**	**2011**	**Combined**	**2010**	**2011**	**Combined**	**2010**	**2011**	**Combined**
FLIP 2010-105L	111.0	104.0	107.5	147.3	136.3	141.8	36.1	23.1	29.6
FLIP 2010-106L	103.2	96.2	99.7	137.3	127.9	132.6	41.2	30.2	35.7
FLIP 2011-43L	106.7	101.3	104.0	140.1	130.1	135.1	41.2	28.5	34.8
FLIP 2011-51L	105.8	96.0	100.9	139.3	128.3	133.8	40.2	28.2	34.2
FLIP 2011-52L	110.0	103.0	106.5	140.1	129.1	134.6	39.6	25.6	32.6
FLIP 2011-54L	102.8	95.8	99.3	137.2	126.2	131.7	41.0	27.0	34.0
FLIP 2011-55L	113.3	100.7	107.0	145.3	131.2	138.2	37.3	21.5	29.4
FLIP 2011-56L	106.2	99.2	102.7	143.2	127.8	135.5	40.3	18.9	29.6
FLIP 2011-57L	97.3	93.0	95.2	134.2	123.4	128.8	38.1	19.7	28.9
ILL 10974	111.3	112.7	112.0	143.8	134.8	139.3	37.1	25.1	31.1
ILL 10975	112.0	105.0	108.5	144.6	133.6	139.1	36.2	24.2	30.2
FLIP 2011-60L	109.3	102.3	105.8	142.2	131.2	136.7	33.1	24.8	29.0
FLIP 2011-61L	107.4	99.0	103.2	139.1	130.8	134.9	40.0	30.0	35.0
Mean	106.3	99.2	102.8	140.8	130.8	135.8	36.7	25.2	31.0
LSD 5% for genotype	4.7	8.4	4.8	4.5	6.4	3.9	5.2	7.2	4.4
LSD 5% for interaction	-	-	ns	-	-	5.5	-	-	6.2

Table S2. Reproductive performance of 35 lentil genotypes grown at Dirab Agriculture Station (Riyadh, Saudi Arabia) in November 2010, December 2011 and combined of the two seasons.

Genotype	Number of Branches/Plant			Number of Seeds/Plant			Seed Yield/Plant (g)		
	2010	2011	Combined	2010	2011	Combined	2010	2011	Combined
ILL 3375	3.9	3.5	3.7	33.4	21.5	27.4	1.2	0.5	0.9
FLIP2009-50L	3.1	2.1	2.6	27.9	13.3	20.6	1.1	0.4	0.8
FLIP 2009-51L	3.6	5.0	4.3	39.6	27.2	33.4	1.2	0.8	1.0
FLIP 2009-52L	4.1	3.8	4.0	25.5	12.0	18.8	1.1	0.7	0.9
FLIP 2009-54L	3.9	2.3	3.1	22.8	20.1	21.5	1.0	0.9	0.9
FLIP 2009-55L	3.4	3.4	3.4	23.3	28.6	26.0	0.9	0.8	0.8
FLIP 2009-56L	2.9	3.3	3.1	26.6	32.0	29.3	1.0	1.3	1.1
FLIP 2009-64L	3.6	4.0	3.8	52.1	33.0	42.6	1.9	1.2	1.5
FLIP 2009-68L	3.9	3.5	3.7	31.5	21.7	26.6	1.1	0.9	1.0
FLIP 2009-69L	3.0	3.5	3.2	41.0	31.0	36.0	1.3	1.0	1.1
FLIP 2009-70L	3.6	3.3	3.5	31.6	18.7	25.2	1.2	0.8	1.0
FLIP 2010-91L	3.4	3.2	3.3	28.7	16.8	22.8	1.1	0.7	0.9
FLIP 2010-94L	4.4	4.2	4.3	37.9	26.3	32.1	1.5	1.2	1.4
FLIP 2010-95L	3.4	3.5	3.5	30.8	21.2	26.0	1.1	0.9	1.0
FLIP 2010-96L	3.1	3.0	3.1	39.5	25.2	32.3	1.2	0.8	1.0
FLIP 2010-97L	3.2	2.9	3.1	33.5	24.0	28.8	1.6	1.3	1.4
FLIP 2010-99L	4.3	3.7	4.0	33.5	22.5	28.0	1.3	1.0	1.2
FLIP 2010-100L	3.9	3.9	3.9	38.6	26.7	32.7	1.3	0.8	1.0
FLIP 2010-101L	2.8	2.6	2.7	44.9	32.9	38.9	1.7	1.3	1.5
FLIP 2010-102L	2.5	2.3	2.4	40.4	29.4	34.9	1.7	1.4	1.5
FLIP 2010-103L	2.6	3.3	3.0	36.7	20.9	28.8	1.4	0.8	1.1
FLIP 2010-104L	2.9	2.9	2.9	47.3	32.6	40.0	1.4	1.2	1.3
FLIP 2010-105L	3.6	3.0	3.3	34.2	23.6	28.9	2.0	1.4	1.7
FLIP 2010-106L	3.4	2.4	2.9	29.3	14.6	22.0	1.4	0.8	1.1

Table S2. *Cont.*

Genotype	Number of Branches/Plant			Number of Seeds/Plant			Seed Yield/Plant (g)		
	**2010**	**2011**	**Combined**	**2010**	**2011**	**Combined**	**2010**	**2011**	**Combined**
FLIP 2011-43L	2.8	2.7	2.8	31.3	20.3	25.8	1.2	0.9	1.0
FLIP 2011-51L	3.6	3.0	3.3	26.0	18.7	22.4	1.3	0.9	1.1
FLIP 2011-52L	2.7	2.1	2.4	26.1	15.1	20.6	1.0	0.7	0.8
FLIP 2011-54L	3.1	3.0	3.0	38.0	26.0	32.0	0.9	0.6	0.8
FLIP 2011-55L	3.5	2.9	3.2	36.0	12.9	24.4	1.4	0.6	1.0
FLIP 2011-56L	3.3	2.2	2.8	30.2	16.3	23.3	1.4	0.7	1.0
FLIP 2011-57L	4.0	2.9	3.5	36.2	17.3	26.8	1.4	0.8	1.1
ILL 10974	2.9	2.9	2.9	32.0	23.4	27.7	1.2	1.0	1.1
ILL 10975	2.8	2.2	2.5	35.1	24.1	29.6	1.2	1.1	1.1
FLIP 2011-60L	3.3	3.4	3.3	31.3	27.3	29.3	1.2	1.0	1.1
FLIP 2011-61L	3.7	3.1	3.4	31.5	23.9	27.7	1.3	1.0	1.2
Mean	3.4	3.1	3.25	33.8	22.9	28.4	1.3	0.9	1.1
LSD 5% for genotype	1.4	1.1	0.9	9.9	9.2	6.7	0.5	0.36	0.3
LSD 5% for interaction	-	-	ns	-	-	ns	-	-	ns

Table S3. Minerals Content of lentil genotypes (on a dry weight basis).

Genotype	Ca	P	K	Mg	Fe	Mn	Cu	Zn
	(mg/100g)							
ILL 3375	79.3	378.2	1032.6	133.8	7.31	2.49	1.26	4.24
FLIP2009-50L	76.4	415.6	993.4	130.7	7.63	2.71	0.87	4.41
FLIP 2009-51L	74.0	286.9	976.2	132.3	7.72	1.68	1.04	4.35
FLIP 2009-52L	64.9	465.3	1061.2	136.5	8.16	1.92	1.37	4.43
FLIP 2009-54L	81.3	546.7	1051.2	140.9	7.45	1.52	1.16	4.03
FLIP 2009-55L	79.7	492.9	985.4	138.3	7.90	2.85	0.92	3.89
FLIP 2009-56L	68.7	365.5	943.0	129.0	7.45	1.38	1.37	3.64
FLIP 2009-64L	76.3	378.6	765.2	135.6	7.00	1.68	1.21	3.61
FLIP 2009-68L	76.0	3.67.4	815.0	138.3	7.31	1.32	1.23	3.44
FLIP 2009-69L	76.4	379.6	859.3	138.6	7.28	1.44	1.35	4.32
FLIP 2009-70L	76.2	375.5	687.4	132.9	6.82	1.42	1.27	3.36
FLIP 2010-91L	79.6	361.9	786.9	139.4	8.57	1.45	1.15	3.82
FLIP 2010-94L	71.3	415.3	674.4	142.6	7.01	1.36	1.26	3.62
FLIP 2010-95L	75.9	395.2	796.9	145.9	6.57	1.35	0.99	2.63
FLIP 2010-96L	75.5	407.9	753.6	150.2	6.71	1.50	0.86	2.74
FLIP 2010-97L	71.5	456.3	848.5	157.3	7.26	1.47	0.89	2.98
FLIP 2010-99L	69.4	531.2	914.4	141.1	7.18	1.50	0.91	3.73
FLIP 2010-100L	77.6	512.7	891.6	148.3	7.55	1.41	1.01	3.79
FLIP 2010-101L	70.2	525.5	885.3	128.9	8.04	1.38	0.97	3.44
FLIP 2010-102L	73.4	538.6	946.4	142.4	7.12	1.42	0.96	2.99
FLIP 2010-103L	72.8	329.6	998.6	126.1	7.13	1.26	1.31	4.38
FLIP 2010-104L	76.0	334.3	899.0	132.5	7.53	1.32	1.28	3.49
FLIP 2010-105L	80.2	335.2	872.1	140.3	7.61	1.30	1.24	4.51
FLIP 2010-106L	84.8	336.4	926.3	147.6	7.09	1.33	0.87	3.83
FLIP 2011-43L	83.5	347.6	981.3	144.9	6.83	1.40	0.98	3.85

Table S3. *Cont.*

Genotype	Ca	P	K	Mg	Fe	Mn	Cu	Zn
	(mg/100g)							
FLIP 2011-51L	70.9	416.2	871.4	132.1	7.28	1.43	1.03	4.06
FLIP 2011-52L	70.5	438.9	869.7	137.3	7.60	1.44	1.25	3.88
FLIP 2011-54L	78.2	428.1	878.7	144.4	8.02	1.32	1.15	4.24
FLIP 2011-55L	71.0	421.0	838.6	137.5	7.51	1.37	1.13	4.09
FLIP 2011-56L	72.6	401.5	776.4	130.4	7.38	1.44	1.31	3.82
FLIP 2011-57L	68.5	345.2	781.9	133.5	8.03	1.37	1.14	3.66
ILL 10974	72.5	438.6	793.0	146.9	7.81	1.31	1.30	4.27
ILL 10975	74.3	451.6	985.2	139.4	7.93	1.42	1.26	3.91
FLIP 2011-60L	75.3	383.2	883.4	131.7	7.76	1.34	1.20	3.28
FLIP 2011-61L	77.5	386.5	991.5	142.8	7.14	1.31	1.18	3.49
Mean ± SE *	74.9 ± 0.7	400.1 ± 16.1	886.1 ± 16.8	138.6 ± 1.2	7.45 ± 0.08	1.53 ± 0.07	1.13 ± 0.03	3.78 ± 0.08

* SE, standard error.

Table S4. Amino acids contents of lentil genotypes (on a dry weight basis). Ala = Alanine; Gly = Glycine; Leu = Leucine; Iso = Isoleucine; Phe = Phenylalanine; Try = Tryptophan; Tyr = Tyrosine; Val = Valine; Pro = Proline.

**Genotype**	**Ala**	**Gly**	**Leu**	**Ile**	**Phe**	**Try**	**Tyr**	**Val**	**Pro**
	**g/kg**								
ILL 3375	10.4	9.8	9.5	4.7	6.7	0.72	6.4	8.5	11.1
FLIP2009-50L	9.6	9.5	7.4	4.2	5.3	0.61	6.6	8.6	11.2
FLIP 2009-51L	11.2	9.8	8.7	5.6	5.6	0.74	6.3	8.6	10.5
FLIP 2009-52L	10.5	8.2	8.0	4.8	5.5	0.83	5.1	7.2	8.3
FLIP 2009-54L	14.6	9.6	6.8	3.4	6.4	0.63	6.5	6.9	11.3
FLIP 2009-55L	10.6	8.1	7.6	3.8	5.7	0.72	6.9	5.3	10.9
FLIP 2009-56L	11.3	9.9	7.9	4.1	5.5	0.69	4.9	7.9	10.5
FLIP 2009-64L	11.6	9.4	8.1	4.2	6.6	0.81	6.5	6.4	9.4
FLIP 2009-68L	10.2	8.1	7.8	3.8	6.1	0.73	5.9	8.4	8.7
FLIP 2009-69L	10.6	8.2	8.4	3.9	5.1	0.69	4.3	5.8	8.4
FLIP 2009-70L	10.4	9.5	9.4	4.7	6.3	0.78	6.1	8.0	7.8
FLIP 2010-91L	10.3	9.8	9.4	5.3	5.5	0.90	3.8	7.6	8.5
FLIP 2010-94L	10.8	8.2	9.0	5.6	5.4	0.92	6.2	8.0	8.1
FLIP 2010-95L	11.2	9.2	8.1	3.8	6.7	0.74	6.9	6.4	8.6
FLIP 2010-96L	9.2	8.9	9.5	4.2	5.8	0.68	6.9	7.3	6.4
FLIP 2010-97L	9.3	9.2	9.1	4.5	5.8	0.89	4.9	6.4	9.5
FLIP 2010-99L	10.3	9.4	9.2	4.4	4.9	0.73	6.9	6.5	6.2
FLIP 2010-100L	10.6	10.1	9.5	5.2	6.5	0.90	6.0	8.0	6.1
FLIP 2010-101L	10.8	10.2	7.7	4.8	6.7	0.76	5.8	9.0	7.3
FLIP 2010-102L	11.5	10.3	8.3	4.3	6.3	0.70	6.2	7.6	8.3
FLIP 2010-103L	10.4	7.2	9.8	3.5	5.8	0.73	5.6	7.9	11.8
FLIP 2010-104L	10.8	9.3	9.2	4.0	5.8	0.81	6.0	6.4	11.5
FLIP 2010-105L	10.4	8.4	7.6	4.1	5.9	0.84	6.2	8.7	11.9
FLIP 2010-106L	9.6	9.8	9.6	3.8	7.6	0.86	6.4	8.2	11.7

Table S4. *Cont.*

Genotype	Ala	Gly	Leu	Ile	Phe	Try	Tyr	Val	Pro
	g/kg								
FLIP 2011-43L	8.2	8.4	9.2	5.3	6.4	0.90	7.4	7.0	9.3
FLIP 2011-51L	10.7	8.8	7.5	4.7	6.1	0.88	7.0	6.5	6.3
FLIP 2011-52L	10.2	8.3	8.4	5.6	6.6	0.89	7.1	6.02	6.4
FLIP 2011-54L	8.6	9.6	8.8	5.3	7.6	0.92	6.8	8.4	8.8
FLIP 2011-55L	11.3	9.5	9.1	4.4	7.2	0.86	4.8	7.5	10.3
FLIP 2011-56L	10.2	9.4	8.2	5.2	7.8	0.87	4.1	7.4	7.8
FLIP 2011-57L	11.9	9.3	8.2	5.4	7.4	0.85	7.2	6.6	7.9
ILL 10974	10.7	7.6	9.3	4.9	7.5	0.81	5.7	8.7	7.5
ILL 10975	10.2	8.4	9.7	4.7	7.5	0.76	5.5	7.9	11.7
FLIP 2011-60L	10.3	8.6	9.3	4.4	7.8	0.64	3.9	5.6	7.7
FLIP 2011-61L	11.0	7.6	9.2	5.3	8.2	0.89	4.6	7.4	8.1
Mean ± SE	10.6 ± 0.2	9.0 ± 0.1	8.6 ± 0.1	4.6 ± 0.1	6.4 ± 0.2	0.79 ± 0.02	5.9 ± 0.2	7.4 ± 0.2	9.0 ± 0.3

Table S5. Amino acids contents of lentil genotypes (on a dry weight basis). Ser = Serine; Thr = Threonine; Glu = Glutamic; Asp = Aspartic; His = Histidine; Cys = Cysteine; Met = Methionine; Lys = Lysine; Arg = Arginine.

**Genotype**	**Ser**	**Thr**	**Glu**	**Asp**	**His**	**Cys**	**Met**	**Lys**	**Arg**
	**g/kg**								
ILL 3375	8.3	5.7	10.5	9.5	3.7	0.62	1.9	4.5	7.2
FLIP2009-50L	7.4	6.3	9.3	10.6	4.5	0.53	1.7	6.4	6.7
FLIP 2009-51L	7.3	4.5	10.9	10.9	4.9	0.47	1.5	6.7	9.1
FLIP 2009-52L	8.7	5.6	11.9	9.6	4.8	0.46	1.6	6.9	9.4
FLIP 2009-54L	7.6	7.5	10.6	10.8	3.9	0.51	1.7	6.8	8.3
FLIP 2009-55L	7.8	6.0	10.3	10.8	5.5	0.58	1.8	6.4	9.4
FLIP 2009-56L	7.4	7.8	11.5	9.7	5.4	0.48	1.6	6.7	8.5
FLIP 2009-64L	9.2	5.9	10.4	9.1	5.4	0.67	1.7	8.6	8.2
FLIP 2009-68L	8.8	7.6	11.2	9.3	5.1	0.64	1.0	8.2	8.8
FLIP 2009-69L	8.6	7.1	10.0	9.4	5.2	0.42	1.2	6.2	10.0
FLIP 2009-70L	9.7	7.1	11.3	9.6	5.9	0.62	1.8	7.5	8.6
FLIP 2010-91L	9.3	6.3	10.0	9.2	4.8	0.43	1.3	8.5	9.1
FLIP 2010-94L	9.9	6.7	9.3	9.4	4.2	0.48	1.1	6.4	10.0
FLIP 2010-95L	8.4	7.9	10.8	9.6	4.4	0.52	1.4	7.6	9.0
FLIP 2010-96L	8.2	6.5	10.2	8.2	5.5	0.49	1.8	8.3	9.8
FLIP 2010-97L	8.9	7.1	11.7	9.2	5.6	0.48	1.7	8.6	9.1
FLIP 2010-99L	9.1	6.9	10.5	10.3	4.8	0.60	1.7	8.4	9.2
FLIP 2010-100L	9.2	4.1	10.2	9.6	4.9	0.59	1.8	8.3	7.0
FLIP 2010-101L	9.3	4.8	10.9	8.9	5.2	0.63	1.5	7.3	6.9
FLIP 2010-102L	9.7	6.8	11.1	10.3	5.9	0.44	1.3	8.6	6.7
FLIP 2010-103L	7.9	4.1	11.7	10.4	3.6	0.62	1.6	5.9	6.6
FLIP 2010-104L	8.9	5.6	11.0	10.0	3.9	0.58	2.1	5.4	7.9
FLIP 2010-105L	8.5	5.4	10.4	10.0	4.7	0.49	1.7	6.0	8.3
FLIP 2010-106L	9.9	6.3	9.8	9.6	4.5	0.42	1.6	6.3	8.6

Table S5. *Cont.*

Genotype	Ser	Thr	Glu	Asp	His	Cys	Met	Lys	Arg
	g/kg								
FLIP 2011-43L	8.4	4.7	11.4	9.2	4.3	0.53	1.6	7.9	7.6
FLIP 2011-51L	9.2	6.9	10.3	9.3	4.4	0.59	0.96	7.3	8.2
FLIP 2011-52L	8.6	4.8	10.9	9.3	5.1	0.60	1.8	7.7	9.5
FLIP 2011-54L	9.2	4.6	10.6	10.4	4.2	0.60	1.9	7.5	8.8
FLIP 2011-55L	8.7	4.5	9.8	10.2	4.9	0.42	1.1	7.9	8.7
FLIP 2011-56L	9.9	5.7	9.1	10.5	4.7	0.57	1.4	7.6	9.5
FLIP 2011-57L	8.0	5.6	10.7	9.7	4.5	0.52	0.85	8.1	9.5
ILL 10974	8.6	5.4	11.0	10.1	4.5	0.49	1.4	7.4	7.3
ILL 10975	8.6	6.9	11.3	10.0	6.1	0.50	1.4	8.5	9.8
FLIP 2011-60L	9.7	6.6	10.8	9.6	5.6	0.62	1.0	8.1	8.7
FLIP 2011-61L	9.0	5.1	9.1	9.3	4.8	0.38	1.1	7.3	8.4
Mean ± SE	8.7 ± 0.1	6.0 ± 0.2	10.6 ± 0.1	9.8 ± 0.1	4.8 ± 0.1	0.53 ± 0.01	1.5 ± 0.1	7.3 ± 0.2	8.5 ± 0.2

Table S6. Antinutritional factors in lentil genotypes (on a dry weight basis).

Genotype	Protease Inhibitors	Tannins	Phytic Acids
	TIA * (mg/g)	mg/g	
ILL 3375	2.48	0.79	0.81
FLIP2009-50L	2.33	0.68	0.87
FLIP 2009-51L	2.64	0.78	0.80
FLIP 2009-52L	2.13	0.67	0.87
FLIP 2009-54L	2.11	0.60	1.01
FLIP 2009-55L	2.48	0.73	0.72
FLIP 2009-56L	2.50	0.74	0.89
FLIP 2009-64L	2.64	0.68	0.92
FLIP 2009-68L	2.66	0.59	0.81
FLIP 2009-69L	2.58	0.65	0.90
FLIP 2009-70L	2.65	0.64	1.06
FLIP 2010-91L	2.49	0.67	0.91
FLIP 2010-94L	2.49	0.69	1.03
FLIP 2010-95L	2.39	0.65	0.95
FLIP 2010-96L	2.61	0.73	0.93
FLIP 2010-97L	2.55	0.75	0.96
FLIP 2010-99L	2.68	0.65	0.94
FLIP 2010-100L	2.72	0.69	0.82
FLIP 2010-101L	*2.54*	*0.68*	*0.87*
FLIP 2010-102L	*2.61*	*0.59*	*0.88*
FLIP 2010-103L	*2.61*	*0.73*	*0.97*
FLIP 2010-104L	*2.56*	*0.78*	*0.76*
FLIP 2010-105L	2.57	0.74	0.92
FLIP 2010-106L	2.47	0.52	0.88
FLIP 2011-43L	2.08	0.70	0.97
FLIP 2011-51L	2.09	0.71	0.96
FLIP 2011-52L	2.69	0.68	1.01
FLIP 2011-54L	2.47	0.63	1.02
FLIP 2011-55L	2.78	0.64	1.07
FLIP 2011-56L	2.72	0.74	1.09
FLIP 2011-57L	2.59	0.54	0.87
ILL 10974	2.73	0.76	0.95
ILL 10975	2.72	0.79	1.01
FLIP 2011-60L	2.43	0.73	1.07
FLIP 2011-61L	2.53	0.62	0.98
Mean ± SE	2.52 ± 0.03	0.69 ± 0.01	0.93 ± 0.02

* TIA, trypsin inhibiting activity.

Table S7. Antioxidant in lentil genotypes.

Genotype	TPC [a] ± SD (mg GAE/g)	TFlC [b] ± SD (mg QE/g)	DPPH [c] ± SD (Vitamin C Equivalents) µg/g
ILL 3375	6.78 ± 0.38	4.12 ± 0.45	13.23 ± 0.22
FLIP2009-50L	12.58 ± 2.11	5.28 ± 0.79	22.38 ± 0.07
FLIP 2009-51L	11.57 ± 2.23	5.22 ± 0.40	15.62 ± 0.14
FLIP 2009-52L	10.38 ± 1.26	4.86 ± 0.42	12.42 ± 0.05
FLIP 2009-54L	15.22 ± 2.34	4.34 ± 0.61	11.27 ± 0.02
FLIP 2009-55L	12.69 ± 1.22	4.67 ± 0.38	16.67 ± 0.12
FLIP 2009-56L	14.77 ± 1.64	6.28 ± 0.22	16.28 ± 0.14
FLIP 2009-64L	18.63 ± 2.15	6.36 ± 0.16	15.37 ± 0.05
FLIP 2009-68L	18.27 ± 2.23	7.12 ± 0.34	13.19 ± 0.04
FLIP 2009-69L	17.45 ± 1.62	6.68 ± 0.14	15.28 ± 0.06
FLIP 2009-70L	14.81 ± 0.71	7.26 ± 0.22	16.86 ± 0.02
FLIP 2010-91L	13.24 ± 1.31	6.76 ± 0.16	11.88 ± 0.11
FLIP 2010-94L	12.64 ± 1.72	5.30 ± 0.13	12.13 ± 0.02
FLIP 2010-95L	20.01 ± 0.60	7.18 ± 0.15	19.12 ± 0.25
FLIP 2010-96L	20.82 ± 1.67	8.56 ± 0.18	18.74 ± 0.16
FLIP 2010-97L	12.43 ± 2.18	5.64 ± 0.12	13.34 ± 0.03
FLIP 2010-99L	19.43 ± 1.54	7.28 ± 0.17	16.25 ± 0.06
FLIP 2010-100L	14.82 ± 0.85	6.32 ± 0.12	14.72 ± 0.02
FLIP 2010-101L	17.35 ± 1.26	6.76 ± 0.15	15.56 ± 0.02
FLIP 2010-102L	9.51 ± 0.30	5.78 ± 0.16	12.19 ± 0.07
FLIP 2010-103L	16.62 ± 0.43	7.22 ± 0.18	18.36 ± 0.12
FLIP 2010-104L	11.53 ± 0.45	6.96 ± 0.11	14.66 ± 0.08
FLIP 2010-105L	7.98 ± 0.42	4.86 ± 0.13	12.54 ± 0.04
FLIP 2010-106L	6.87 ± 0.62	4.22 ± 0.16	13.21 ± 0.05
FLIP 2011-43L	8.69 ± 0.84	4.74 ± 0.12	12.84 ± 0.06
FLIP 2011-51L	9.16 ± 0.55	5.36 ± 0.18	11.66 ± 0.07
FLIP 2011-52L	6.45 ± 0.26	4.36 ± 0.06	12.32 ± 0.06
FLIP 2011-54L	7.68 ± 0.28	4.46 ± 0.08	10.61 ± 0.08
FLIP 2011-55L	7.82 ± 0.42	4.26 ± 0.11	12.23 ± 0.06
FLIP 2011-56L	18.22 ± 0.55	8.32 ± 0.06	17.64 ± 0.05
FLIP 2011-57L	17.42 ± 0.86	7.66 ± 0.14	18.14 ± 0.03
ILL 10974	20.52 ± 2.42	8.92 ± 0.24	23.26 ± 0.04
ILL 10975	6.45 ± 0.27	4.18 ± 0.05	15.11 ± 0.05
FLIP 2011-60L	14.24 ± 0.64	6.25 ± 0.17	15.67 ± 0.04
FLIP 2011-61L	18.29 ± 0.98	7.52 ± 0.04	17.46 ± 0.12

[a] Total phenolic components calculated as Galic Acid Equivalent/g seed; [b] total flavenoid content (mg Quercetin Equivalent/g seed); [c] 2-Diphenyl-1-picryl hydrazyl radical (µg/g); SD, standard division.

Table S8. Proximate Analysis of Lentil Genotypes Seeds (on a dry weight basis).

Genotype	Crude Protein	Moisture	Ash	Fats	Carbohydrate
			%		
ILL 3375	27.2	12.4	2.67	1.03	42.7
FLIP 2009-50L	26.7	12.1	2.78	1.01	46.2
FLIP 2009-51L	28.4	12.1	2.44	1.13	44.7
FLIP 2009-52L	27.9	12.6	2.39	1.01	44.8
FLIP 2009-54L	26.5	12.4	2.66	1.14	45.0
FLIP 2009-55L	28.8	12.6	2.48	1.12	43.7
FLIP 2009-56L	26.3	12.4	2.60	0.79	46.5
FLIP 2009-64L	25.9	12.5	2.49	1.02	43.1
FLIP 2009-68L	26.7	12.5	2.52	0.98	44.8
FLIP 2009-69L	28.9	12.6	2.67	0.97	43.5
FLIP 2009-70L	25.3	13.1	2.53	0.89	40.9
FLIP 2010-91L	26.4	11.9	2.39	1.03	43.2
FLIP 2010-94L	26.7	12.1	2.57	1.19	40.9
FLIP 2010-95L	26.8	11.6	2.62	1.04	40.8
FLIP 2010-96L	29.2	12.0	2.65	0.99	47.1
FLIP 2010-97L	26.6	12.8	2.54	0.97	41.3
FLIP 2010-99L	27.2	13.3	2.47	0.76	43.6
FLIP 2010-100L	28.5	12.7	2.59	0.72	41.4
FLIP 2010-101L	29.1	12.5	2.50	0.89	41.6
FLIP 2010-102L	25.8	13.0	2.46	0.83	39.7
FLIP 2010-103L	25.3	13.4	2.89	1.16	41.5
FLIP 2010-104L	27.4	12.9	2.63	1.12	39.5
FLIP 2010-105L	28.3	12.7	2.67	1.00	43.8
FLIP 2010-106L	28.4	12.5	2.62	1.19	46.7
FLIP 2011-43L	29.0	12.4	2.63	0.95	46.4
FLIP 2011-51L	25.3	12.3	2.71	1.12	46.2
FLIP 2011-52L	25.7	12.7	2.68	1.14	45.9
FLIP 2011-54L	25.5	13.3	2.61	1.13	44.9
FLIP 2011-55L	27.4	13.6	2.78	0.97	47.0
FLIP 2011-56L	28.0	13.1	2.66	0.89	45.3
FLIP 2011-57L	29.3	12.8	2.63	0.84	46.2
ILL 10974	29.1	12.6	2.61	1.00	44.7
ILL 10975	28.4	12.7	2.83	0.97	45.2
FLIP 2011-60L	27.7	12.9	2.61	0.96	44.9
FLIP 2011-61L	27.4	13.0	2.58	1.02	46.2
Mean ± SE	27.4 ± 0.2	12.6 ± 0.1	2.61 ± 0.02	1.00 ± 0.02	44.0 ± 0.4

Figure S1. Jaccard similarity coefficient values for pairwise comparison of 35 lentil genotypes using Sequence Related Amplified Polymorphism (SRAP) primers.

	ILL-3375	FLIP2009-50L	FLIP2009-51L	FLIP2009-52L	FLIP2009-54L	FLIP2009-55L	FLIP2009-56L	FLIP2009-46L	FLIP2009-68L	FLIP2009-69L	FLIP2009-70L	FLIP2010-91L	FLIP2010-94L	FLIP2010-95L	FLIP2010-96L	FLIP2010-97L	FLIP2010-99L	FLIP2010-100L	FLIP2010-101L	FLIP2010-102L	FLIP2010-103L	FLIP2010-104L	FLIP2010-105L	FLIP2010-106L	FLIP2011-43L	FLIP2011-51L	FLIP2011-52L	FLIP2011-54L	FLIP2011-55L	FLIP2011-56L	FLIP2011-57L	ILL10974	ILL10975	FLIP2011-60L	FLIP2011-61L
ILL-3375	1																																		
FLIP2009-50L	0.30	1.00																																	
FLIP2009-51L	0.00	0.21	1.00																																
FLIP2009-52L	0.29	0.41	0.34	1.00																															
FLIP2009-54L	0.18	0.33	0.38	0.35	1.00																														
FLIP2009-55L	0.25	0.43	0.29	0.50	0.34	1.00																													
FLIP2009-56L	0.12	0.29	0.23	0.27	0.30	0.38	1.00																												
FLIP2009-46L	0.24	0.23	0.14	0.29	0.25	0.27	0.28	1.00																											
FLIP2009-68L	0.41	0.47	0.12	0.40	0.29	0.39	0.35	0.34	1.00																										
FLIP2009-69L	0.32	0.31	0.18	0.36	0.27	0.31	0.23	0.27	0.35	1.00																									
FLIP2009-70L	0.17	0.24	0.36	0.31	0.35	0.33	0.22	0.24	0.23	0.26	1.00																								
FLIP2010-91L	0.32	0.38	0.26	0.39	0.39	0.36	0.28	0.30	0.40	0.40	0.25	1.00																							
FLIP2010-94L	0.00	0.17	0.30	0.18	0.16	0.18	0.28	0.12	0.18	0.20	0.14	0.17	1.00																						
FLIP2010-95L	0.21	0.33	0.19	0.33	0.29	0.29	0.34	0.34	0.39	0.25	0.17	0.29	0.22	1.00																					
FLIP2010-96L	0.27	0.42	0.44	0.54	0.38	0.46	0.23	0.21	0.38	0.34	0.46	0.35	0.23	0.28	1.00																				
FLIP2010-97L	0.36	0.52	0.24	0.52	0.36	0.48	0.36	0.29	0.51	0.44	0.28	0.45	0.21	0.40	0.47	1.00																			
FLIP2010-99L	0.20	0.30	0.22	0.46	0.36	0.50	0.35	0.34	0.37	0.36	0.27	0.39	0.23	0.39	0.34	0.46	1.00																		
FLIP2010-100L	0.28	0.42	0.31	0.49	0.43	0.50	0.29	0.28	0.37	0.37	0.44	0.40	0.17	0.31	0.48	0.45	0.40	1.00																	
FLIP2010-101L	0.26	0.37	0.35	0.46	0.33	0.39	0.21	0.24	0.30	0.34	0.74	0.34	0.12	0.18	0.59	0.38	0.23	0.54	1.00																
FLIP2010-102L	0.30	0.36	0.22	0.42	0.40	0.36	0.21	0.28	0.40	0.33	0.28	0.46	0.13	0.27	0.38	0.40	0.30	0.43	0.34	1.00															
FLIP2010-103L	0.10	0.20	0.15	0.24	0.20	0.21	0.25	0.24	0.22	0.21	0.16	0.19	0.28	0.26	0.20	0.20	0.31	0.17	0.14	0.09	1.00														
FLIP2010-104L	0.40	0.48	0.14	0.44	0.30	0.44	0.36	0.30	0.58	0.36	0.24	0.41	0.22	0.37	0.40	0.58	0.39	0.43	0.31	0.42	0.21	1.00													
FLIP2010-105L	0.24	0.41	0.26	0.44	0.33	0.33	0.27	0.27	0.41	0.44	0.34	0.38	0.23	0.31	0.45	0.43	0.37	0.44	0.39	0.32	0.34	0.40	1.00												
FLIP2010-106L	0.35	0.33	0.16	0.39	0.29	0.35	0.25	0.29	0.48	0.40	0.27	0.43	0.16	0.24	0.34	0.38	0.36	0.38	0.35	0.36	0.22	0.44	0.39	1.00											
FLIP2011-43L	0.22	0.31	0.28	0.37	0.31	0.33	0.20	0.24	0.26	0.34	0.54	0.36	0.15	0.19	0.42	0.32	0.29	0.51	0.70	0.33	0.20	0.24	0.41	0.34	1.00										
FLIP2011-51L	0.37	0.41	0.19	0.49	0.35	0.45	0.33	0.36	0.50	0.46	0.31	0.51	0.21	0.33	0.45	0.53	0.45	0.48	0.38	0.47	0.22	0.56	0.47	0.49	0.34	1.00									
FLIP2011-52L	0.29	0.42	0.22	0.38	0.35	0.28	0.21	0.28	0.44	0.45	0.27	0.43	0.23	0.29	0.42	0.43	0.38	0.37	0.34	0.34	0.32	0.39	0.52	0.37	0.35	0.44	1.00								
FLIP2011-54L	0.34	0.41	0.18	0.43	0.30	0.40	0.34	0.29	0.50	0.47	0.28	0.40	0.24	0.36	0.39	0.52	0.40	0.43	0.35	0.37	0.20	0.49	0.42	0.44	0.33	0.55	0.40	1.00							
FLIP2011-55L	0.08	0.10	0.00	0.10	0.08	0.10	0.11	0.13	0.13	0.10	0.05	0.05	0.06	0.13	0.05	0.14	0.08	0.08	0.00	0.09	0.00	0.11	0.08	0.09	0.01	0.12	0.10	0.12	1.00						
FLIP2011-56L	0.26	0.38	0.26	0.42	0.38	0.43	0.44	0.34	0.48	0.41	0.29	0.43	0.25	0.37	0.35	0.57	0.46	0.45	0.31	0.38	0.22	0.47	0.43	0.40	0.31	0.52	0.40	0.52	0.11	1.00					
FLIP2011-57L	0.11	0.15	0.11	0.14	0.21	0.17	0.31	0.19	0.31	0.17	0.09	0.19	0.24	0.27	0.09	0.22	0.22	0.15	0.04	0.16	0.13	0.24	0.14	0.17	0.06	0.22	0.19	0.21	0.19	0.23	1.00				
ILL10974	0.32	0.38	0.36	0.48	0.39	0.43	0.29	0.31	0.42	0.33	0.41	0.39	0.16	0.29	0.49	0.49	0.34	0.49	0.54	0.42	0.18	0.42	0.38	0.40	0.40	0.45	0.36	0.40	0.09	0.43	0.17	1.00			
ILL10975	0.28	0.42	0.35	0.58	0.39	0.59	0.34	0.33	0.45	0.38	0.40	0.41	0.19	0.34	0.51	0.50	0.43	0.59	0.44	0.42	0.18	0.47	0.44	0.45	0.39	0.51	0.39	0.47	0.08	0.47	0.16	0.56	1.00		
FLIP2011-60L	0.23	0.30	0.16	0.34	0.40	0.28	0.32	0.37	0.42	0.34	0.26	0.39	0.16	0.31	0.28	0.37	0.39	0.32	0.20	0.32	0.32	0.39	0.39	0.41	0.22	0.45	0.41	0.34	0.12	0.39	0.25	0.35	0.38	1.00	
FLIP2011-61L	0.23	0.41	0.31	0.43	0.33	0.41	0.29	0.23	0.41	0.48	0.39	0.42	0.28	0.30	0.49	0.45	0.40	0.50	0.39	0.32	0.28	0.42	0.64	0.38	0.44	0.51	0.62	0.52	0.00	0.49	0.11	0.35	0.49	0.31	1.00

Figure S2. Jaccard similarity coefficient values for pairwise comparison of 35 lentil genotypes using Amplified Fragment Length Polymorphism (AFLP).

	ILL-3375	FLIP2009-50L	FLIP2009-51L	FLIP2009-52L	FLIP2009-54L	FLIP2009-55L	FLIP2009-56L	FLIP2009-46L	FLIP2009-68L	FLIP2009-69L	FLIP2009-70L	FLIP2010-91L	FLIP2010-94L	FLIP2010-95L	FLIP2010-96L	FLIP2010-97L	FLIP2010-99L	FLIP2010-100L
ILL-3375	1.00																	
FLIP2009-50L	0.55	1.00																
FLIP2009-51L	0.54	0.46	1.00															
FLIP2009-52L	0.56	0.54	0.55	1.00														
FLIP2009-54L	0.42	0.30	0.28	0.31	1.00													
FLIP2009-55L	0.30	0.28	0.34	0.29	0.40	1.00												
FLIP2009-56L	0.41	0.33	0.33	0.31	0.47	0.46	1.00											
FLIP2009-46L	0.20	0.17	0.19	0.17	0.37	0.32	0.37	1.00										
FLIP2009-68L	0.10	0.08	0.11	0.08	0.17	0.22	0.24	0.30	1.00									
FLIP2009-69L	0.57	0.55	0.49	0.60	0.32	0.29	0.33	0.19	0.10	1.00								
FLIP2009-70L	0.18	0.15	0.19	0.16	0.15	0.22	0.24	0.14	0.29	0.17	1.00							
FLIP2010-91L	0.57	0.42	0.46	0.51	0.43	0.36	0.41	0.27	0.13	0.54	0.19	1.00						
FLIP2010-94L	0.12	0.10	0.18	0.10	0.19	0.28	0.26	0.19	0.38	0.09	0.38	0.13	1.00					
FLIP2010-95L	0.44	0.44	0.36	0.36	0.45	0.38	0.39	0.29	0.13	0.43	0.11	0.40	0.15	1.00				
FLIP2010-96L	0.29	0.32	0.39	0.29	0.12	0.30	0.20	0.10	0.15	0.36	0.30	0.30	0.30	0.22	1.00			
FLIP2010-97L	0.39	0.33	0.42	0.38	0.28	0.33	0.27	0.29	0.16	0.35	0.12	0.34	0.15	0.52	0.35	1.00		
FLIP2010-99L	0.49	0.48	0.47	0.49	0.41	0.49	0.45	0.25	0.14	0.53	0.19	0.46	0.18	0.50	0.36	0.44	1.00	
FLIP2010-100L	0.39	0.36	0.44	0.41	0.14	0.27	0.23	0.09	0.17	0.36	0.33	0.29	0.19	0.19	0.38	0.27	0.36	1.00
FLIP2010-101L	0.37	0.38	0.36	0.33	0.09	0.17	0.16	0.10	0.12	0.40	0.30	0.30	0.13	0.15	0.39	0.21	0.28	0.64
FLIP2010-102L	0.32	0.28	0.33	0.29	0.37	0.40	0.45	0.37	0.27	0.28	0.15	0.42	0.16	0.42	0.21	0.47	0.41	0.28
FLIP2010-103L	0.39	0.40	0.36	0.34	0.45	0.32	0.42	0.33	0.14	0.46	0.19	0.42	0.13	0.38	0.24	0.26	0.46	0.16
FLIP2010-104L	0.42	0.40	0.39	0.40	0.40	0.46	0.49	0.30	0.18	0.47	0.19	0.49	0.15	0.49	0.25	0.34	0.49	0.20
FLIP2010-105L	0.20	0.18	0.26	0.27	0.12	0.28	0.15	0.18	0.24	0.30	0.34	0.32	0.23	0.21	0.41	0.30	0.23	0.33
FLIP2010-106L	0.47	0.40	0.47	0.43	0.38	0.36	0.42	0.33	0.13	0.44	0.21	0.60	0.17	0.49	0.29	0.43	0.49	0.28
FLIP2011-43L	0.53	0.37	0.41	0.36	0.38	0.37	0.35	0.24	0.11	0.41	0.30	0.49	0.14	0.42	0.29	0.39	0.38	0.26
FLIP2011-51L	0.50	0.56	0.47	0.57	0.34	0.34	0.35	0.19	0.11	0.60	0.23	0.51	0.12	0.40	0.35	0.33	0.54	0.36
FLIP2011-52L	0.58	0.44	0.57	0.55	0.32	0.32	0.36	0.20	0.10	0.54	0.18	0.48	0.14	0.44	0.33	0.39	0.48	0.32
FLIP2011-54L	0.48	0.45	0.48	0.41	0.32	0.37	0.34	0.24	0.12	0.43	0.21	0.41	0.23	0.53	0.38	0.50	0.58	0.34
FLIP2011-55L	0.27	0.27	0.30	0.30	0.38	0.36	0.40	0.42	0.19	0.29	0.10	0.36	0.15	0.38	0.13	0.26	0.40	0.15
FLIP2011-56L	0.38	0.38	0.41	0.36	0.40	0.49	0.44	0.27	0.18	0.41	0.21	0.38	0.26	0.47	0.32	0.36	0.68	0.29
FLIP2011-57L	0.45	0.38	0.41	0.40	0.44	0.38	0.58	0.34	0.20	0.40	0.31	0.51	0.17	0.35	0.22	0.28	0.49	0.27
ILL10974	0.48	0.47	0.50	0.43	0.22	0.30	0.31	0.25	0.16	0.50	0.22	0.45	0.11	0.31	0.31	0.41	0.45	0.51
ILL10975	0.44	0.44	0.48	0.43	0.26	0.30	0.33	0.22	0.14	0.49	0.20	0.43	0.11	0.32	0.29	0.38	0.44	0.44
FLIP2011-60L	0.29	0.28	0.33	0.29	0.22	0.27	0.24	0.33	0.24	0.31	0.20	0.37	0.19	0.30	0.31	0.35	0.30	0.34
FLIP2011-61L	0.17	0.16	0.23	0.15	0.12	0.20	0.12	0.18	0.26	0.17	0.14	0.18	0.24	0.27	0.31	0.29	0.20	0.25

	FLIP2010-101L	FLIP2010-102L	FLIP2010-103L	FLIP2010-104L	FLIP2010-105L	FLIP2010-106L	FLIP2011-43L	FLIP2011-51L	FLIP2011-52L	FLIP2011-54L	FLIP2011-55L	FLIP2011-56L	FLIP2011-57L	ILL10974	ILL10975	FLIP2011-60L	FLIP2011-61L
FLIP2010-101L	1.00																
FLIP2010-102L	0.19	1.00															
FLIP2010-103L	0.22	0.27	1.00														
FLIP2010-104L	0.18	0.40	0.52	1.00													
FLIP2010-105L	0.32	0.26	0.17	0.32	1.00												
FLIP2010-106L	0.25	0.42	0.49	0.56	0.27	1.00											
FLIP2011-43L	0.28	0.33	0.41	0.44	0.26	0.46	1.00										
FLIP2011-51L	0.36	0.33	0.44	0.48	0.25	0.47	0.47	1.00									
FLIP2011-52L	0.27	0.29	0.39	0.53	0.25	0.55	0.46	0.54	1.00								
FLIP2011-54L	0.27	0.36	0.36	0.45	0.30	0.52	0.43	0.41	0.53	1.00							
FLIP2011-55L	0.11	0.38	0.34	0.38	0.22	0.34	0.28	0.30	0.32	0.35	1.00						
FLIP2011-56L	0.19	0.40	0.43	0.49	0.28	0.47	0.34	0.40	0.41	0.56	0.43	1.00					
FLIP2011-57L	0.23	0.32	0.60	0.51	0.23	0.59	0.46	0.43	0.44	0.41	0.40	0.42	1.00				
ILL10974	0.45	0.35	0.32	0.36	0.32	0.44	0.37	0.37	0.40	0.45	0.25	0.38	0.46	1			
ILL10975	0.43	0.33	0.33	0.43	0.28	0.47	0.35	0.37	0.38	0.44	0.25	0.46	0.39	0.64	1.00		
FLIP2011-60L	0.29	0.45	0.23	0.35	0.41	0.45	0.27	0.29	0.31	0.36	0.29	0.35	0.27	0.42	0.37	1.00	
FLIP2011-61L	0.21	0.28	0.12	0.24	0.36	0.24	0.16	0.14	0.22	0.25	0.15	0.24	0.11	0.23	0.23	0.42	1

© 2013 by the authors; licensee MDPI, Basel, Switzerland. This article is an open access article distributed under the terms and conditions of the Creative Commons Attribution license (http://creativecommons.org/licenses/by/3.0/).
